# Research on Optimizing Forming Accuracy in Food 3D Printing Based on Temperature–Pressure Dual Closed-Loop Control

**DOI:** 10.3390/mi16101156

**Published:** 2025-10-12

**Authors:** Junhua Wang, Hao Cao, Jianan Shen, Xu Duan, Yanwei Xu, Tancheng Xie, Ruijie Gu

**Affiliations:** 1 School of Mechanical and Electrical Engineering, Henan University of Science and Technology, Luoyang 471003, China; wangjh@haust.edu.cn (J.W.); 16637950853@163.com (H.C.);; 2 College of Food and Bioengineering, Henan University of Science and Technology, Luoyang 471000, China; 3 Henan Intelligent Manufacturing Equipment Engineering Technology Research Center, Luoyang 471003, China; 4 Henan Engineering Laboratory of Intelligent Numerical Control Equipment, Luoyang 471003, China

**Keywords:** starch-based printing material, food 3D printing system, Bang-Bang and PID hybrid control, adaptive fuzzy PID extrusion pressure control

## Abstract

In this paper, a new 3D printing system based on temperature–pressure double closed-loop collaborative control is proposed to solve the problem of 3D printing accuracy of starch food. The rapid and accurate adjustment of the nozzle temperature is realized by the hybrid control of Bang-Bang and PID, and the extrusion pressure is optimized in real time by combining the adaptive fuzzy PID algorithm, which effectively reduces the influence from the change of material rheological properties and external interference. The experimental results show that the printing accuracy of the system is up to 98% at 40 °C, the pressure fluctuation is reduced by 80%, and the molding accuracy of complex structures is improved to 97%, which significantly improves the over-extrusion and under-extrusion, and provides an effective solution for stable and high-precision printing of high-viscosity food materials.

## 1. Introduction

Food 3D printing, as an emerging digital manufacturing technology, demonstrates immense potential in areas such as personalized nutrition and the preparation of complex-structured foods [1,2]. However, the core bottleneck in transitioning from concept to application lies in the difficulty of achieving stable, reliable, high-precision forming [3]. Extrusion forming precision is fundamentally a control issue of “depositing the correct amount of material at the correct time and in the correct location.” First, the rheological properties of printed materials constitute the fundamental internal factor affecting forming precision. Unlike homogeneous, stable industrial printing materials, food printing materials are predominantly multiphase, multicomponent non-Newtonian fluids whose viscosity significantly depends on shear rate and temperature [4,5]. However, most current research treats materials as static systems, neglecting the real-time rheological changes induced by temperature fluctuations and shear history during printing [6]. Second, control lag and fluctuations in the pressure extrusion process constitute key external factors causing precision defects. Ideal stable extrusion requires constant matching between the material flow rate and nozzle movement speed. Extrusion pressure fluctuations directly manifest as flow instability, leading to over-extrusion or under-extrusion [7]. Traditional open-loop or simple PID control systems exhibit response delays to extrusion pressure disturbances, failing to adapt to dynamic demands arising from path geometry and material property variations during printing [8]. When printing corners, the system’s “overshoot-oscillation” phenomenon becomes particularly pronounced, severely compromising geometric feature fidelity.

Currently, to enhance extrusion precision in food 3D printing, researchers primarily focus on three approaches: material modification, process optimization, and control system development. Jiwon In et al. [9] improved print fidelity, shape retention, and extrusion properties by adding pectin to gelatin, thereby increasing the gel’s print adaptability. Z Liu et al. [10] enhanced the shear-thinning behavior of gel systems by incorporating starch and xanthan gum, thereby improving extrusion properties, recovery capability, and self-supporting 3D structures of printed materials. V Prithviraj et al. [11] optimized rheological properties of rice flour paste by adding coarse sugar, leading to enhanced printability and stability of final products. Yang F et al. [12] optimized baking dough printing parameters, determining the optimal combination as filament diameter 2.30 mm, nozzle speed 25 mm/s, nozzle diameter 2.0 mm, and nozzle height 2.40 mm, significantly improving product forming precision. Bareen M A et al. [13] optimized printing parameters for thermoacid-coagulated milk semi-solids combined with polyols, identifying optimal conditions of flow rate 28 mm^3^/s, nozzle diameter 1.0 mm, and layer height 67% for excellent print adaptability. Jiao X et al. [14] optimized printing parameters for corn starch-based materials, finding optimal printability when gelatinization temperature ranged from 75–85 °C and starch content was 15–20%. These studies focused on enhancing the static rheological properties of food printing materials through additives like thickeners and pectin, or experimentally identifying fixed “optimal” printing parameters. While effective to some extent, these approaches remain inherently open-loop and passive. In the realm of control systems, Kim N P et al. [15] achieved stable extrusion of high-viscosity food printing materials in 3D printing by optimizing piston extrusion. Martínez-Monzó J et al. [16] developed a thermostatic control system to maintain the nozzle at 30 °C, ensuring smooth extrusion of mashed potatoes. Umeda T et al. [17] introduced pressure sensors and employed a PID controller to stabilize extrusion pressure, discovering that stable printing was achievable within a pumpkin flake content range of 20.0–28.6 wt%. Existing studies typically address temperature or pressure control in isolation, and the algorithms employed lack sufficient capability to handle nonlinear time-varying systems. Most critically, few investigations systematically consider temperature and pressure control as an integrated whole, resulting in significant limitations when addressing the time-varying rheological properties of materials and external disturbances during printing.

To address the aforementioned issues, this study proposes and implements a novel dual-closed-loop temperature–pressure cooperative control architecture. Rather than simply superimposing temperature and pressure control, this architecture establishes a cascaded optimization logic: a temperature Bang-Bang and PID composite control system first creates a stable processing environment for the material, approximating an ideal rheological state. Building upon this foundation, an adaptive fuzzy PID pressure control system further suppresses external disturbances to achieve precise flow control. This “temperature-first, pressure-second” collaborative paradigm provides a systematic solution for addressing multi-variable coupling challenges.

## 2. System Design and Control Strategy

### 2.1. Mechanical System Design for Food 3D Printers

**Figure 1:**
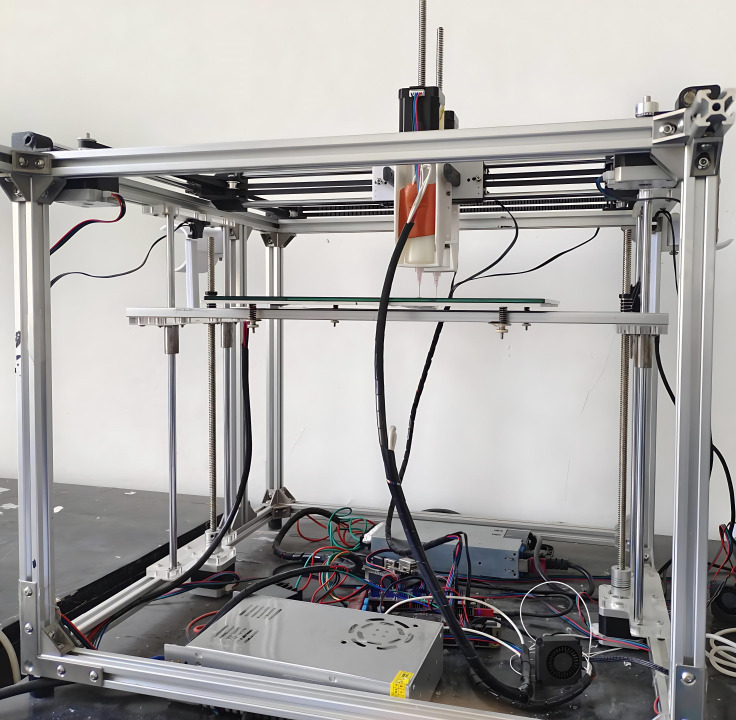
Food 3D Printer.

The overall structure of a food 3D printer primarily consists of an end-effector, material feeding mechanism, control system, and actuation structure, as shown in Figure 1. The end-effector comprises an extrusion motor, piston, extrusion barrel, and nozzle. The extrusion motor transmits rotational motion to the piston within the extrusion barrel via a lead screw, enabling the extrusion process. Heaters and temperature sensors are installed on both the end effector and printing platform. A composite control algorithm enables precise temperature regulation, enhancing model print quality. The actuation system employs a stable hybrid-CoreXY structure, where X and Y motors synergistically drive belts to move sliders within the XY plane. These sliders feature linear bearings that operate via rolling friction on shafts. On the Z-axis, the print bed connects to a lead screw via a nut. Motor rotation drives the lead screw, enabling vertical movement of the print bed. This structure delivers high printing stability and precision while offering a simple, cost-effective design. It is well-suited for high-viscosity food printing applications and provides a stable foundation for dual closed-loop temperature–pressure control.

The printer’s travel range is set to 300 mm × 300 mm × 300 mm, thereby defining the printer’s overall frame. The printer’s basic parameters are shown in Table 1.

### 2.2. Overall Architecture of the Control System

**Figure 2:**
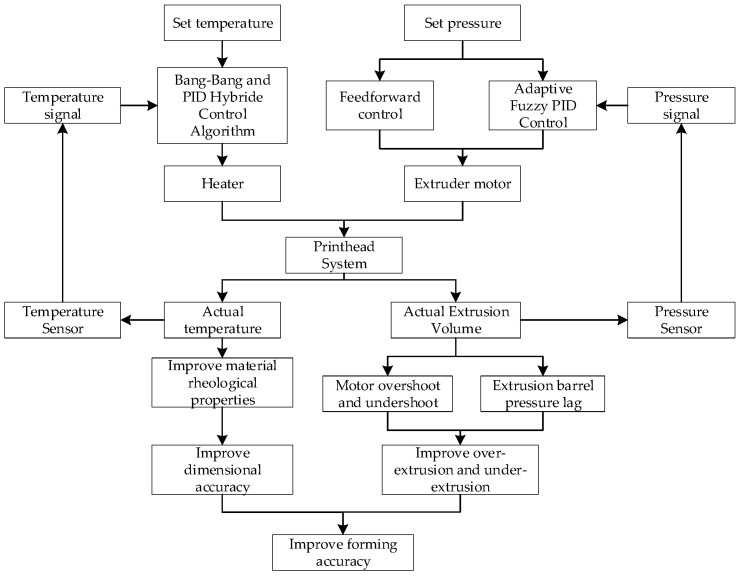
Overall Architecture Diagram of the Control System.

This study employs a dual-closed-loop temperature–pressure control architecture, with the overall control system structure shown in Figure 2. The temperature closed loop utilizes a Bang-Bang and PID composite control algorithm to regulate heater power, stabilizing the nozzle temperature at the setpoint to provide a consistent rheological environment for food printing materials. The pressure closed-loop employs a feedforward-feedback composite control strategy. The feedforward controller compensates based on motion commands, while the feedback controller adjusts according to pressure sensor readings. The combined outputs of both controllers jointly regulate the extrusion motor speed, thereby achieving precise extrusion flow control.

### 2.3. Design of a Temperature Control System Based on a Bang-Bang and PID Hybrid Approach

During the 3D printing of starch-based foods, forming precision is influenced by multiple factors, including the levelness of the heated bed, slicing parameter settings, and nozzle temperature. Among these, nozzle temperature significantly impacts the rheological behavior of the printing material. Research indicates that appropriately raising the temperature can stabilize the rheological properties of starch-based materials, extending their printable time window [18,19]. However, during actual heating, the nozzle temperature control system often exhibits issues such as excessive overshoot and slow response. These dynamic instabilities directly compromise the consistency of material rheological performance, thereby degrading print quality. Therefore, achieving precise control of the nozzle temperature is crucial for ensuring printing accuracy.

**Figure 3:**
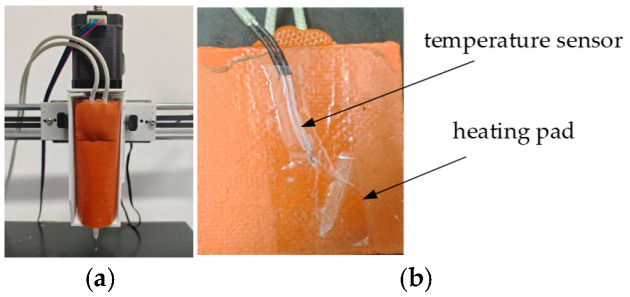
Printhead and Temperature Sensor Installation Diagram. (**a**) Overall Structure of the Nozzle; (**b**) Temperature Sensor Installation Location.

To enable real-time monitoring and regulation of nozzle temperature, this system incorporates custom heating elements mounted on the outer surface of the nozzle. NTC temperature sensors are positioned on the inner side of these heating elements to achieve closed-loop control of nozzle temperature. This configuration effectively minimizes the impact of thermal inertia while enhancing the responsiveness of temperature detection and control. The specific installation structure is illustrated in Figure 3.

To address the overshoot and response lag issues in the aforementioned temperature control, this study proposes a composite Bang-Bang and PID control strategy. Bang-Bang control is a simple, fast-responding nonlinear control method where the output switches between only two preset states. This strategy determines the output state based on the sign of the error between the actual system temperature and the setpoint, rather than the magnitude of the error. Consequently, it exhibits rapid initial response, significantly reducing the time required for the system to approach the target temperature and enhancing the speed of the dynamic process. Its control expression is:

(1) $$u(k)=\{\begin{array}{c} u_{\max},|e(k)|>\varepsilon \\ 0,|e(k)|\leq \varepsilon \end{array}$$

where *u*(*k*) is the current controller output; *u_max_* is the maximum output value; *e*(*k*) is the error; *ε* is the set threshold.

**Figure 4:**
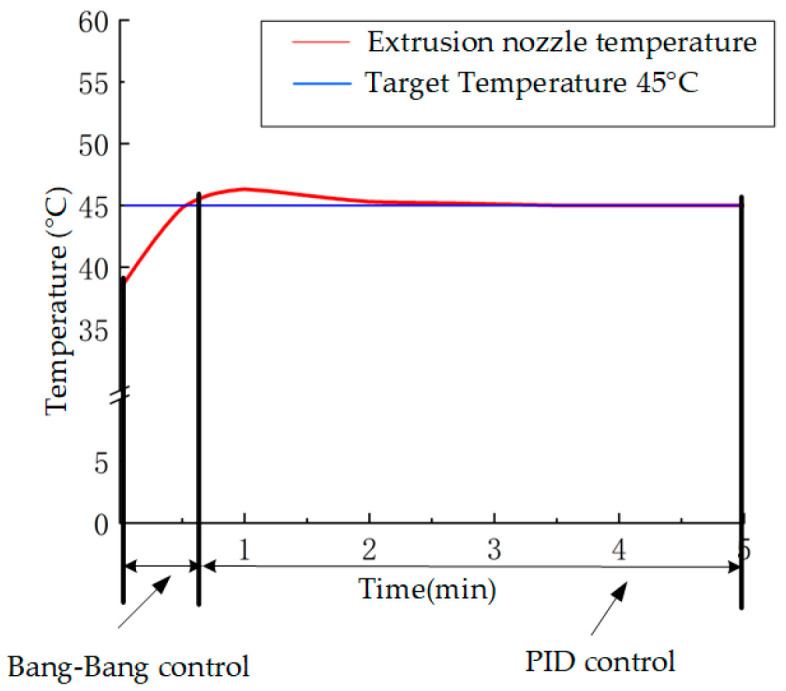
Hybrid Control Distribution Chart.

In this system, *ε* is introduced as a threshold. When the absolute value of the deviation *e*(*k*) between the target temperature and the actual temperature exceeds *ε*, the system enters Bang-Bang control mode, where the heater output is set to the maximum value *u*(*k*). Conversely, when *e*(*k*) is less than *ε*, the system enters PID control mode for precise continuous regulation. The composite control distribution is shown in Figure 4. The PID control expression is:

(2) $$u(k)=K_{p}e(k)+K_{i}\int_{0}^{k}e(\tau )d\tau +K_{d}\frac{de(k)}{dk}$$

where *K_p_* is the proportional coefficient; *K_i_* is the integral coefficient and *K_d_* is the derivative coefficient.

**Figure 5:**
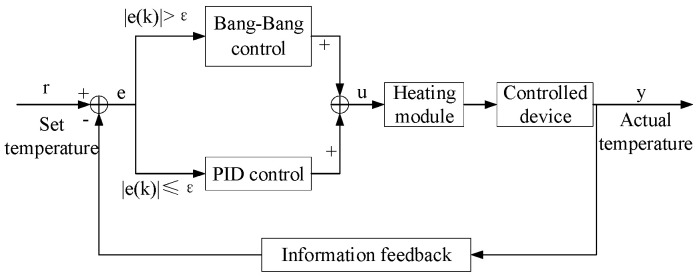
Temperature Control System Block Diagram.

During the PID control process, the system drives the heating element through the heating module. The temperature sensor continuously monitors the nozzle temperature, converting the temperature signal into a voltage signal via an analog-to-digital converter for feedback to the controller. The controller continuously monitors temperature changes, calculates the error between the current temperature and the setpoint, and dynamically adjusts the PWM duty cycle based on the PID algorithm. This precisely controls the heating power output, enabling the system’s actual temperature to gradually converge toward the target temperature. Once the temperature reaches the setpoint, the system enters a steady-state regulation phase, effectively suppressing overshoot and minimizing steady-state error to achieve high-precision temperature control. The overall structure of this temperature control system is shown in Figure 5.

### 2.4. Design of a Pressure Control System Based on a Feedforward-Feedback Hybrid Structure

Pressure control in the extrusion process is inherently a complex system characterized by significant dynamic lag and time-varying disturbances. To address this challenge, this study employs a feedforward-feedback hybrid control architecture. First, pressure feedforward control is used to mitigate the deterministic dynamic lag of the system. An approximate mathematical model of the extrusion process is established. Compensation values are pre-calculated based on the geometric information of the printing path and superimposed onto the control command. This mitigates over-extrusion and under-extrusion caused by motor acceleration or deceleration. Secondly, adaptive fuzzy PID control suppresses uncertain disturbances and model errors. Due to the time-varying rheological properties of food materials and the difficulty in establishing precise mathematical models, residual errors inevitably exist in feedforward compensation. The adaptive fuzzy PID, functioning as a feedback controller, dynamically adjusts PID parameters by continuously monitoring pressure deviations and their rates of change. This enables online compensation for disturbances caused by unpredictable factors such as variations in material viscosity and fluctuations in friction resistance, ensuring system stability and robustness under diverse operating conditions.

#### 2.4.1. Mathematical Model Development

Pressure pre-control is a system that adjusts material flow rate by regulating internal pressure within the extrusion barrel. This is achieved through nozzles that apply instantaneous forces at material extrusion and stop positions. However, due to the complexity of non-Newtonian fluids, current linear models represent a simplified first-order approximation. Adaptive control is employed to overcome model uncertainty. To quantitatively describe this process, this study makes several reasonable assumptions:

(1) Neglecting secondary factors such as gravity and surface tension, focus solely on the “compression/resistance” of material within the extrusion path and its “discharge” at the nozzle.
(2) The extrusion path can be regarded as a chamber with equivalent “spring” characteristics, meaning that when the extruder stepper motor applies a unit material inflow, the internal pressure *p*(*t)* within the chamber changes accordingly; as pressure increases, the actual flow rate at the nozzle also changes.
(3) Assume the nozzle flow rate *Q_out_*(*t*) approximates a linear relationship with pressure *p*(*t*), or more generally a monotonic function relationship (here, for ease of derivation, a linear approximation is adopted).

Establish a fluid dynamics model based on volume conservation and the “elasticity” assumption: when instantaneous inflow exceeds outflow, the internal pressure in the extrusion barrel rises; conversely, it decreases.

(3) $$\frac{dp(t)}{dt}=C\left[Q_{\mathrm{in}}(t)-Q_{\mathrm{out}}(t)\right]$$

where *Q_in_*(*t*) is the ideal volumetric flow rate extruded by the nozzle; *Q_out_*(*t*) iss the actual volumetric flow rate extruded by the nozzle; *P*(*t*) is the instantaneous pressure inside the extrusion barrel; *C* is the resistance coefficient of material extrusion.

Assuming the nozzle discharge rate is linearly proportional to pressure, we have:

(4) $$Q_{\mathrm{out}}(t)=\alpha p(t)$$

where *α* is a proportional coefficient determined by the nozzle geometry and the rheological properties of the material.

Substituting into Equations (3) yields:

(5) $$\frac{dp(t)}{dt}=C\left[Q_{\mathrm{in}}(t)-\alpha p(t)\right]$$

(6) $$\frac{dp(t)}{dt}+(\alpha C)p(t)=CQ_{\mathrm{in}}(t)$$

(7) $$\tau =\frac{1}{\alpha C}$$

where *ꚍ* represents the characteristic time constant of the system.

When *Q_in_*(*t*) changes abruptly, pressure *p*(*t*) requires a time scale of *ꚍ* to amplify or attenuate. The actual flow rate *Q_out_*(*t*) from the nozzle exhibits this same lag, meaning *Q_in_*(*t*) and *Q_out_*(*t*) are not synchronized but filtered by this first-order system.

To reduce this “energy lag,” a ‘feedforward’ or “compensation” term is added to the extrusion command. This causes the system to extrude more material in advance when acceleration is needed, and to extrude less or retract material in advance when deceleration is required. Consequently, the actual flow rate at the nozzle more closely matches the desired target flow rate.

The pressure lead formula is:

(8) $$E_{\mathrm{command}}(t)=E_{\mathrm{basic}}(t)+k_{\mathrm{pa}}\frac{d}{dt}[E_{\mathrm{basic}}(t)]$$

where *E_command_*(*t*) is the step command without pressure compensation; *K_pa_* is the pressure advance coefficient; and *d*[*E_basic_(t)*]/*dt* is the rate of change of the extrusion rate.

This formula constitutes the feedforward compensator for this system. Based on the extrusion rate command *E_command_*(*t*) and its rate of change *dE*/*dt* provided by the motion planning module, the controller calculates the pre-compensation amount using the feedforward coefficient *K_pa_*. This amount is directly added to the output to counteract foreseeable dynamic lag, thereby reducing overshoot or undershoot.

#### 2.4.2. Adaptive Fuzzy PID Pressure Control Structure Design

During the dynamic extrusion of starch paste, direct control of extrusion pressure is difficult to achieve. Typically, pressure on the material is regulated indirectly by adjusting the feed rate or displacement of the screw. Since food printing materials are predominantly non-Newtonian fluids, they exhibit complex rheological properties during extrusion. System parameters such as viscosity and flow behavior demonstrate significant time-varying characteristics and uncertainty, making it difficult to establish precise mathematical models. Consequently, traditional control systems often have limited applicability in such scenarios. To address these challenges, this study developed an adaptive fuzzy PID control system. This system can identify system states online and dynamically adjust control parameters, thereby achieving rapid and stable extrusion pressure control. This ensures consistent and reliable extrusion speeds.

The traditional PID control law, as shown in Equation (9):

(9) $$u(t)=K_{p}\left[e(t)+\frac{1}{T_{i}}\int_{0}^{t}e(\tau )d\tau +T_{d}\frac{de(t)}{dt}\right]$$

where *K_p_* is the proportional coefficient; *T_i_* is the integral time constant; *T_d_* is the derivative time constant.

Discretizing (9) yields:

(10) $$u(k)=K_{p}e(k)+K_{i}T\sum_{j=0}^{k}e(j)+\frac{K_{d}}{T}[e(k)-e(k-1)]$$

where *T* is the sampling period; *k* is the sampled signal; *e*(*k*) is the deviation value of the *k*th sampling.

Thus, pressure acquisition during the forming process is discrete, necessitating the development of an incremental PID control algorithm:

(11) $$\Delta u(k)=K_{P}\Delta e(k)+K_{i}e(k)+K_{d}\left[\Delta e(k)-\Delta e(k-1)\right]$$

where Δ*e*(*k*) represents the difference between the signal deviation at time *k* and the signal deviation at time *k* − 1.

**Figure 6:**
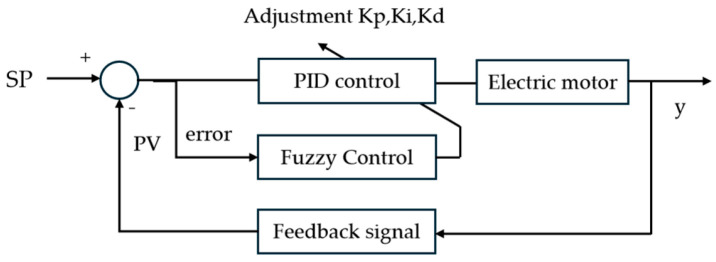
Pressure Control System Block Diagram.

The pressure control system structure is shown in Figure 6. The overall structure primarily consists of two parts: traditional PID regulation and fuzzy control. The fuzzy control section adopts a two-input, three-output configuration. The fuzzy adaptive PID control dynamically adjusts *K_p_*, *K_i_*, and *K_d_* by performing fuzzy reasoning calculations on the error between the set pressure *SP* and the feedback pressure *PV*, as well as the rate of change *K_ec_* over relative time. This modifies the feed distance of the push rod to achieve pressure regulation. The adaptive fuzzy PID controller operates within the feedback loop. The primary function of the feedback loop is to overcome inaccuracies in the feedforward model and unknown disturbances such as variations in material properties. This further reduces steady-state error and enhances the system’s robustness.

#### 2.4.3. Blurred Design

In the design of the fuzzy controller, the error *K_e_* between the pressure setpoint (*SP*) and the feedback pressure (*PV*) along with its rate of change *K_ec_* are selected as input variables for fuzzy reasoning. The parameter increments Δ*K_p_*, Δ*K_i_*, and Δ*K_d_* from the traditional PID controller serve as output variables for fuzzy reasoning. Based on experimental data and system requirements, the fuzzy domains for input variables *K_e_* and *K_ec_* are both set to [−6, 6]. The fuzzy domains for output variables Δ*K_p_*, Δ*K_i_*, and Δ*K_d_* are set to [−0.3, 0.3], [−0.06, 0.06], and [−6, 6], respectively. Each fuzzy variable is divided into seven fuzzy levels, with linguistic variables defined as: Negative Large (NB), Negative Medium (NM), Negative Small (NS), Zero (ZO), Positive Small (PS), Positive Medium (PM), and Positive Large (PB). Thus, the fuzzy subsets are {NB, NM, NS, ZO, PS, PM, PB}. Regarding membership function selection, the Z-type function is applied to the “NB” subset in each domain, the S-type function to the “PB” subset, and the triangular function to all other subsets. Fuzzy inference rules were formulated based on the influence mechanism of PID parameters on system dynamics and practical control experience. The fuzzy rule for Δ*Kp* is shown in Table 2. The designed fuzzy domains establish mapping relationships with actual physical quantities through quantization factors (*K_e_*, *K_ec_*) and proportional factors (*K_p_*, *K_i_*, *K_d_*). These factors were initially set using the Ziegler-Nichols engineering tuning method and further optimized based on control performance during printed experiments to ensure optimal overall system performance.

In fuzzy reasoning, the “AND” operation of fuzzy sets employs a minimization operator, the ‘OR’ operation uses a maximization operator, and the “implication” operation follows the minimization rule. The merging of multiple fuzzy rules is achieved by taking the union of their output fuzzy subsets. The defuzzification process adopts the maximum average membership degree method to obtain precise control outputs.

Let *u* denote the defuzzified precise output value. The membership function of set *A* in domain *U* is *A*(*u*), where *u* ∈ *U*.

*A*(*uj*) *= max*(*A*(*u*)), *j* = 1, 2, …, *n*. The membership degree corresponding to the *n* points is taken to its maximum value, then:

(12) $$u_{m}=\frac{\sum_{j=1}^{n}u_{j}}{n}$$

Fuzzy control employs control rules expressed in the form of IF...THEN...

## 3. Materials and Methods

### 3.1. Equipment

This food 3D printer is an independently developed device. Its overall frame is constructed from aluminum extrusions (Jiangsu RuiZhou Supply Chain Co., Ltd., Wuxi, China). The printer’s motor drivers and control mainboard (Shenzhen Biqu Technology Co., Ltd., Shenzhen, China) are the silent driver TMC2209 and the BIGTREETECH Octopus V1.0 supporting 8-channel stepper motors, respectively. Both the drive motor and extruder motor utilize 42-step stepper motors. (Hampus Co., Ltd., Guangzhou, China). The heating element for the nozzle temperature control is custom-made to fit the nozzle dimensions. The temperature sensor is an NTC 100K thermistor (Jiangsu Xiaochuangxin Thermal Energy Technology Co., Ltd., Wuxi, China). The power supply (Delta Electronics, Inc., Dongguan, China) is a switching power supply rated at 12V. These components collectively form the printer, which achieves three-dimensional shaping of starch-based materials through the coordinated operation of its various modules.

### 3.2. Rheological Characterization and Preparation of Printing Materials

The rheological properties of printing materials are critical factors affecting the quality of 3D printed parts. During extrusion, materials must exhibit low viscosity to ensure smooth passage through the nozzle, while simultaneously possessing sufficient viscoelasticity post-extrusion to maintain structural shape and achieve stable deposition on the printing platform [20]. In food 3D printing, the extrusion mechanism drives material flow directionally through pressure, exhibiting typical pseudoplastic fluid behavior [21,22]. A key characteristic of such fluids is a significant decrease in apparent viscosity with increasing shear rate, manifesting as reduced viscosity at higher extrusion speeds, which facilitates material flow and forming.

Corn starch was purchased from Hubei Yucheng E-commerce Co., Ltd. (Jingmen, China). All printing materials had a total volume mass fraction of 100 mL. Printing materials with varying viscosities were prepared at different gelatinization temperatures for comparative analysis of printing precision.

Corn starch was weighed and mixed with deionized water at a mass ratio of 3:17 to prepare the printing material. This mixture was then placed in an 80 °C water bath and gelatinized for 16 min, 14 min, and 12 min, respectively, to obtain starch paste printing materials of varying viscosities. Stirring was performed throughout gelatinization to prevent solidification into particles that could clog the nozzle. After gelatinization, the material was stirred for 2 min to homogenize it. It was then cooled to room temperature and finally loaded into the extruder barrel of the printer, ready for printing.

### 3.3. Experimental Protocol and Printing Parameters

To validate the impact of the pressure control system and temperature control system designed for this research institute’s food 3D printer on product printing accuracy, the following experiments were conducted:

(1) Printing was performed with pressure control enabled and disabled, respectively, using a standard cube with 20 mm edges as the print model. The effects of pressure control on model surface accuracy were analyzed by comparing over-extrusion and under-extrusion on the first layer, surface layer, and sides of the model.
(2) Printing was conducted with the extrusion barrel temperature set to 35 °C, 40 °C, and 45 °C. A standard rectangular prism (30 mm × 20 mm × 10 mm) served as the print model. The physical length, width, and height of the printed prism were measured, and dimensional errors under different temperatures were compared and analyzed.

Printed products included cuboids and cubes. Over-extrusion and under-extrusion occurring under different pressure control conditions were marked, and evaluated by comparing the affected parts.

For products printed with materials of varying viscosities at different extruder temperatures, the length, width, and height of the cuboids were measured using a vernier caliper. 

The most critical parameters in 3D printing are print speed, nozzle diameter, nozzle height above the print bed, extruder barrel temperature, infill density, and pressure. These parameters determine whether the printing material can be uniformly extruded onto the print bed. For this experiment, the print speed was set to 12 mm/s, with an inner nozzle diameter of 0.84 mm and an outer diameter of 1.26 mm. The nozzle height above the print bed was set at 0.8 mm. Barrel temperatures were tested at 35 °C, 40 °C, and 45 °C, with a fill rate of 80% and pressures of 0.5 MPa and 1 MPa. Printing was conducted using the food 3D printer designed by this research institute.

### 3.4. Data Measurement and Analysis Methods

The fundamental requirement for food 3D printers is to coordinate printing speed and extrusion speed to achieve precise printing. To validate the printing accuracy of the food 3D printer designed in this study, three printing materials with different viscosities were printed in triplicate, yielding a total of 27 samples. Photographs were taken sequentially, and physical dimensions were measured using vernier calipers. The measured data were grouped by viscosity and analyzed at different temperature gradients within each viscosity group. Physical dimensions at varying temperatures within the same viscosity group were examined, with print accuracy serving as the final criterion for evaluating each printed product’s quality. The formula for calculating print accuracy is as follows:

(13) $$Printing\;accuracy(\%)=\frac{\left(1-\frac{\left|L_{a1}-L_{a}\right|}{L_{a}}\right)+\left(1-\frac{\left|L_{b1}-L_{b}\right|}{L_{b}}\right)+\left(1-\frac{\left|H_{c1}-H_{c}\right|}{H_{c}}\right)}{3}\times 100$$

where *L_a_*_1_ is the length; *L_a_* is the set length; *L_b_*_1_ is the width; *L_b_* is the set width; *H_c_*_1_ is the height; *L_c_* is the set height.

## 4. Results and Discussion

Precise temperature control is essential for stable pressure operation. Therefore, we first analyze the decisive influence of temperature on printing accuracy and evaluate the performance of the temperature controller itself. Only at the optimal temperature point do the material’s rheological properties enter an optimal range. At this point, the linear approximation of the pressure feedforward model is most effective, while the material’s time-varying characteristics are minimal, significantly reducing the burden on the feedback controller. Temperature instability directly causes drastic fluctuations in material flow resistance, leading to pressure control system failure. The dual-closed-loop architecture in this study suppresses the primary internal disturbance through the temperature loop, thereby creating conditions for the pressure loop to manage external and dynamic disturbances.

### 4.1. Analysis of Temperature Effects on Printing Accuracy and Control Performance

**Figure 7:**
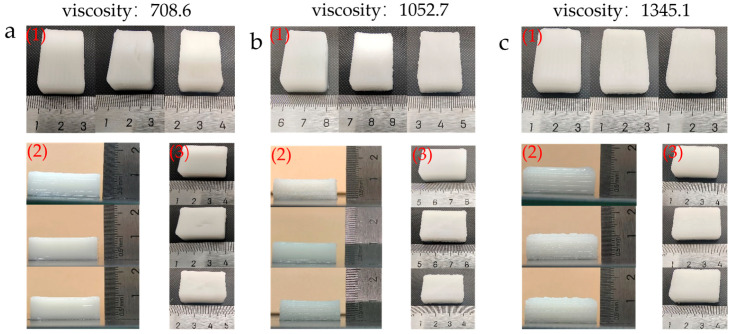
Comparison of printed objects at different viscosities: (**a**) material viscosity 708.6 Pa·s, (**b**) material viscosity 1052.7 Pa·s, (**c**) material viscosity 1345.1 Pa·s. Note: For each group, nozzle temperatures from left to right are room temperature, 40 °C, and 45 °C.

The physical dimension measurements of the 3D-printed food products are shown in Figure 7. Analysis indicates that printed products in the high-viscosity material group exhibit significant deviations from the target dimensions, predominantly manifesting as positive dimensional errors. In contrast, the low-viscosity material group demonstrates higher dimensional stability with smaller errors, resulting in products closer to the designed geometric dimensions. This phenomenon may be closely related to the influence of starch paste viscosity on extrusion flowability: higher viscosity increases extrusion resistance, leading to insufficient rebound and excessive diffusion of the material after extrusion, thereby causing dimensional deviations. As a key process parameter for viscosity control, temperature adjustment within a certain range can effectively reduce material yield stress and improve extrudability. However, excessively high temperatures may accelerate moisture evaporation and starch retrogradation, diminishing fluidity and consequently hindering improvements in printing precision.

**Figure 8:**
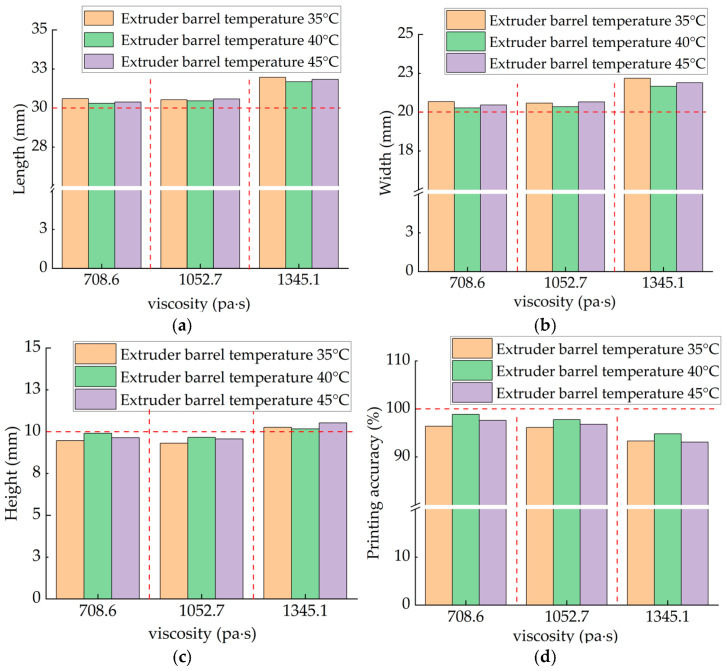
Comparison of Printed Object Measurements at Different Viscosities. (**a**) Comparison of Printed Object Length at Different Nozzle Temperatures; (**b**) Comparison of Printed Object Width at Different Nozzle Temperatures; (**c**) Comparison of Height in Printed Objects at Different Nozzle Temperatures; (**d**) Comparison of Solid Object Printing Accuracy at Different Nozzle Temperatures.

Figure 8 shows the print accuracy comparison of food 3D printers under different viscosity materials. Experimental results indicate that printing temperature significantly affects the forming accuracy of starch-based materials. Across different viscosity groups, when the extrusion barrel temperature is 40 °C, printed parts exhibit the closest dimensions to the standard model in length, width, and height, demonstrating optimal dimensional accuracy. Particularly in the low-viscosity material group, the minimum printing error reached 1.12%. When temperatures deviated from this value, printing accuracy showed a noticeable decline, indicating that 40 °C can be regarded as the rheological inflection point for this material system. At this temperature, the material simultaneously exhibits suitable fluidity and viscoelasticity, facilitating uniform deposition and shape retention of the extruded filament. From a micro-mechanism perspective, 40 °C likely approximates the gelatinization onset temperature for starch-based materials. At this point, polymer chains undergo initial unfolding and enhanced hydration, optimizing the material’s viscoelasticity and extrusion continuity while improving interlayer bonding strength. The results indicate that printing temperature significantly influences forming accuracy by regulating material rheological behavior. A sensitive critical point exists near 40 °C for this material system: temperature deviations exceeding ±5 °C cause significant accuracy degradation. This reflects a critical rheological transition occurring within this range, demanding high precision and stability in temperature control.

**Figure 9:**
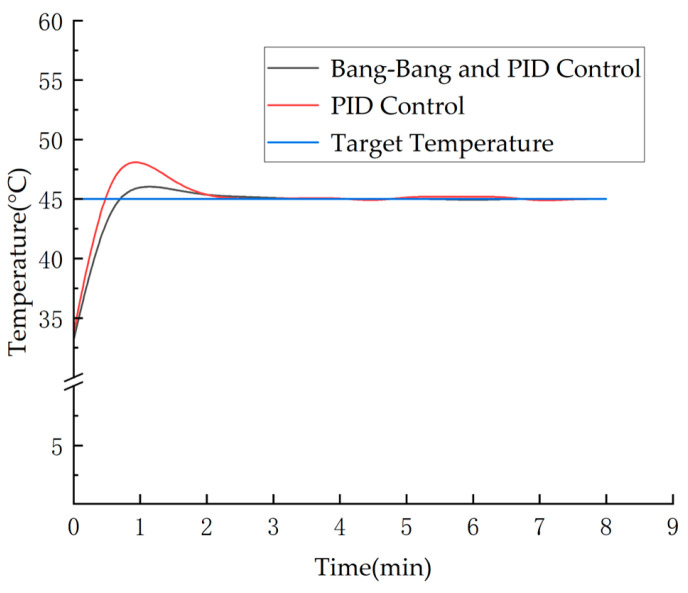
Real-time Monitoring Results of Temperature Control Effectiveness.

In summary, the performance of a temperature control system must strictly limit temperature fluctuations near the operating point to an extremely narrow range. The Bang-Bang and PID composite control strategy employed in this study successfully confined nozzle temperature variations to within ±1 °C, as demonstrated by the real-time temperature monitoring results shown in Figure 9. This provides a reliable foundation for achieving stable, repeatable, high-precision printing near the optimal setpoint of 40 °C. In contrast, when using only a traditional PID controller, temperature fluctuations exceeded ±8 °C. This inability to effectively maintain operation near this rheologically sensitive critical point resulted in significantly degraded print quality and poor repeatability.

### 4.2. Improvement of Print Topography Through Pressure Control and System Response Analysis

**Figure 10:**
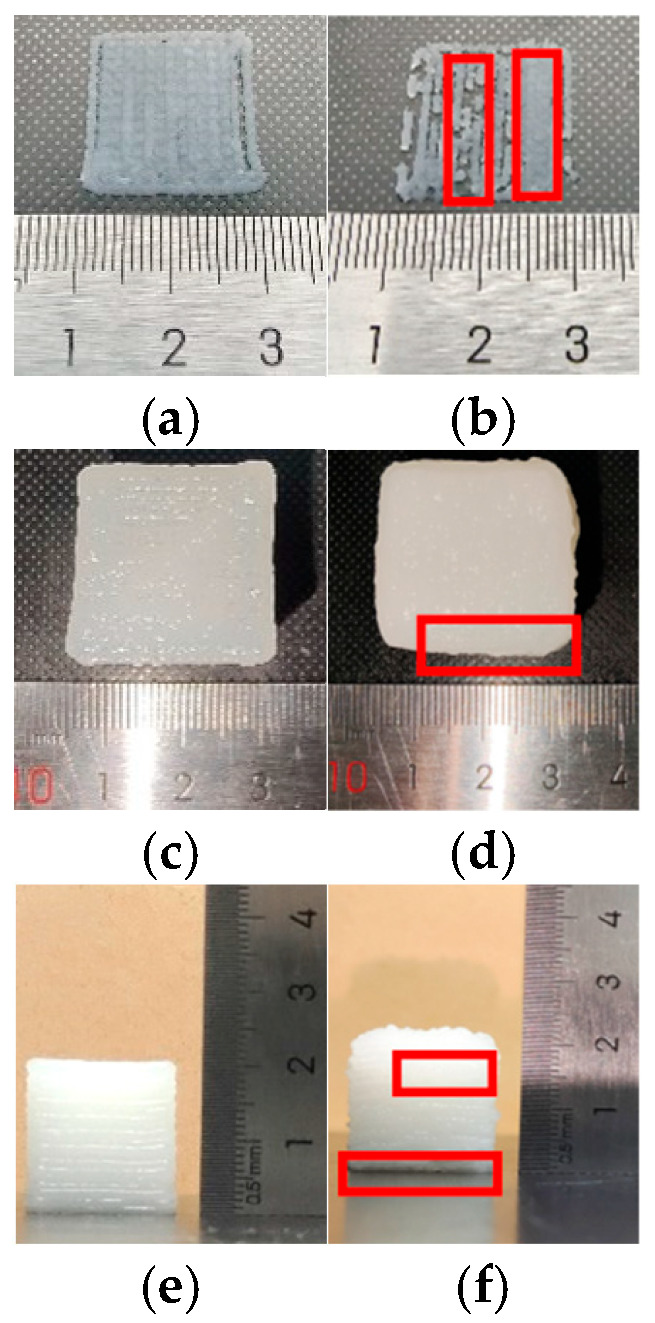
Comparison of Printed Object Morphology. (**a**) Printed Product 1: Surface Topography of the First Layer; (**b**) Printed Product 2: Surface Topography of the First Layer; (**c**) Surface Topography of Printed Product 1; (**d**) Surface Topography of Printed Product 2; (**e**) Printed Product 1 Side Profile; (**f**) Printed Product 2 Side Profile.

To evaluate the impact of pressure feedback control on the surface profile accuracy of printed products, this study prepared two cubic specimens for comparative analysis: Product 1 was printed with pressure feedback control enabled, while Product 2 was printed without it disabled. Their surface profiles are compared in Figure 10.

Comparing the first-layer printing results in Figure 10a,b reveals that without pressure control, the nozzle exhibits highly unstable extrusion during the initial printing stage. The left side of the first layer exhibits “under-extrusion,” while the right side shows “over-extrusion,” as indicated by the red box in Figure 10b. The reason for this is as follows: When the extrusion speed varies with the scanning speed of the printing platform, the extrusion force cannot rapidly reach a steady state. This leads to a lag in material flow response, resulting in uneven extrusion traces on the first layer and severely compromising the surface quality of the printed blank. In Product 1, however, the introduction of pressure feedback control enables the extrusion force to stabilize quickly, significantly improving the uniformity of first-layer extrusion and thereby enhancing the quality of the formed surface.

Further comparison of surface topographies in Figure 10c,d reveals noticeable material over-accumulation in certain areas of Product 2, as indicated by the red box in Figure 10d. This phenomenon stems from overshoot during stepper motor start/stop cycles, causing instantaneous extrusion volume spikes that trigger “dribbling” of extruded filament. In contrast, Product 1 shows no significant accumulation at the same locations, indicating that the adaptive fuzzy PID control strategy effectively suppresses motor overshoot and loss of synchronization. This ensures synchronized extrusion volume with the motion trajectory, thereby enhancing overall molding quality.

Finally, the side profiles shown in Figure 10e,f reveal that Product 2 exhibits prolonged pressure build-up during extrusion and low material filling efficiency, resulting in an uneven internal structure with noticeable extrusion defects, excessive extrusion in the upper layer and insufficient extrusion in the lower layer, as indicated by the red box in Figure 10f. In contrast, Product 1 achieves rapid and stable extrusion pressure through its pressure feedback system, significantly improving filling efficiency and effectively enhancing the overall uniformity and topographical precision of the printed structure.

**Figure 11:**
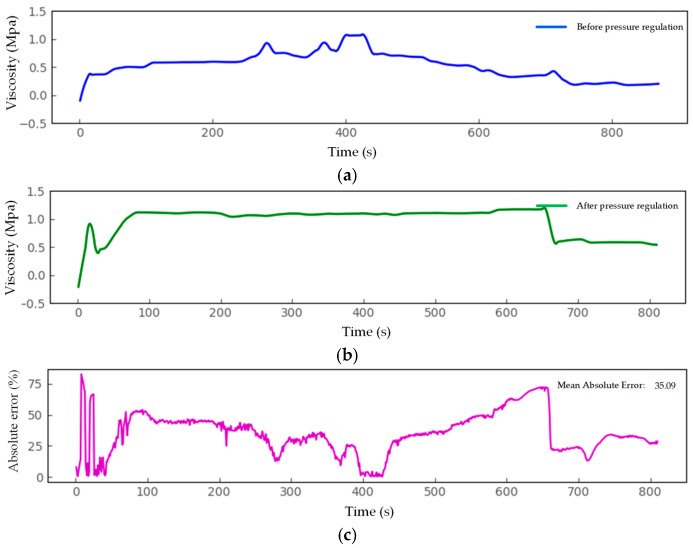
Real-time Monitoring Results of Pressure Control Effectiveness. (**a**) Pressure data monitored when pressure feedback control is not enabled. (**b**) Pressure data monitored when pressure feedback control is enabled. (**c**) Comparison of Average Absolute Pressure Error with and without Pressure Feedback Control Enabled.

In summary, pressure control is a critical method for enhancing nozzle extrusion stability and improving the dimensional accuracy of printed products. Its core lies in achieving rapid response and stable maintenance of material pressure by the piston, thereby ensuring uniform material extrusion. Real-time pressure monitoring results during printing are shown in Figure 11: Figure 11a displays the pressure curve without pressure feedback control (Product 2), exhibiting significant fluctuations; Figure 11b shows the pressure response after enabling pressure feedback control (Product 1), demonstrating excellent stability.

Without control, the system exhibits severe pressure fluctuations. While pressure feedforward control alone can partially suppress pressure peaks at corners, its ability to compensate for sustained disturbances remains limited. After introducing pressure feedback control, system pressure fluctuations decreased significantly, with the standard deviation dropping from 0.537 MPa to 0.103 MPa, and response speed also markedly improved. By adjusting motor speed in real-time via an adaptive fuzzy PID algorithm, the system response time was reduced by 50%, stabilizing in just 30 s. This effectively compensated for extrusion lag caused by material thixotropy. Simultaneously, the pressure peak suppression rate at corners reached 80%, essentially eliminating overshoot and oscillation in the extrusion motor. This significantly mitigated under-extrusion and over-extrusion defects during printing.

These results demonstrate that feedforward control effectively overcomes the system’s dynamic lag, while adaptive feedback control substantially enhances suppression against random disturbances and model uncertainty. Their synergistic interaction constitutes the key mechanism for achieving high-precision extrusion control.

### 4.3. Complex Structure Printing Validation

**Figure 12:**
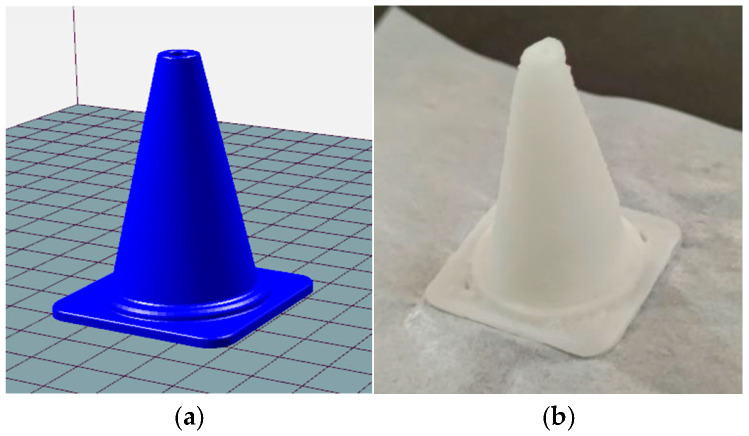
Conical structure printed after enabling the dual control system. (**a**) Conical structure; (**b**) Printed product.

To validate the forming accuracy of the dual-feedback control system in complex structure printing, a starch paste with a viscosity of 708.6 Pa·s was used as the printing material. Printing experiments of conical structures were conducted under conditions of a constant extrusion barrel temperature of 40 °C, and pressure control was enabled. The printed specimen is shown in Figure 12. The base edge length and height of the conical structure were measured using a vernier caliper. Printing accuracy was calculated based on Formula (13), revealing a comprehensive dimensional accuracy of 97%. The actual dimensional error was controlled within ±0.5 mm, meeting the equipment requirements for high-precision food printing [23]. Morphological observation revealed a smooth surface on the printed part with no noticeable under-extrusion or over-extrusion defects, indicating excellent stability in the extrusion process. These results confirm that the proposed temperature–pressure dual feedback control system effectively overcomes precision limitations in 3D printing of multi-viscosity food materials, significantly enhancing the forming quality of complex structures.

## 5. Conclusions

To enhance the forming precision of starch-based food 3D printing, this study designed and developed a food 3D printing system based on dual closed-loop temperature–pressure control and systematically validated its performance. Key findings are as follows:

(1) At a printing temperature of 40 °C, the printing accuracy of the low-viscosity material group reached 98%, significantly higher than the accuracy at 35 °C (with an error of 3.6%). Both high- and medium-viscosity material groups showed increasing accuracy with rising temperature, while low-viscosity materials exhibited a 1% accuracy decline at 45 °C. This indicates 40 °C as the optimal printing temperature, where the material resides within a “rheological window”, offering both favorable extrusion flow and structural retention capabilities.
(2) After introducing pressure feedback control, the standard deviation of extrusion pressure decreased by 0.434 MPa, and system response time shortened by 50%, effectively compensating for extrusion lag caused by material thixotropy. At path corners, pressure peaks were significantly suppressed by 80%, essentially eliminating overshoot and oscillation issues common in traditional PID control.
(3) With dual-loop temperature–pressure control, dimensional accuracy for complex conical structures improved to 97%, while “under-extrusion” and “over-extrusion” phenomena were largely eliminated. This demonstrates the system’s ability to significantly enhance forming quality and process stability when printing complex structures using multi-viscosity food materials.

## Figures and Tables

**Table 1:** The basic parameters of the food 3D printer.

Parameter	Value
Effective travel range (mm)	300 × 300 × 300
Positioning accuracy (mm)	0.01~0.06
Printing speed (mm∙s^−1^)	0~300
Printer dimensions (mm)	500 × 500 × 500
Nozzle diameter (mm)	0.8~1.6

**Table 2:** Δ*Kp* Fuzzy Rule Control Table.

*Ke_c_*								
		NB	NM	NS	ZO	PS	PM	PB
*K_e_*	NB	PB	PB	PM	PM	PS	ZO	ZO
	NM	PB	PB	PM	PS	PS	ZO	NS
	NS	PM	PM	PM	PS	ZO	NS	NS
	ZO	PM	PM	PS	ZO	NS	NM	NM
	PS	PS	PS	ZO	NS	NS	NM	NM
	PM	PS	ZO	NS	NM	NM	NM	NB
	PB	ZO	ZO	NM	NM	NM	NB	NB

## Data Availability

The original contributions presented in this study are included in the article. Further inquiries can be directed to the corresponding authors.
